# Association of post-smoking cessation changes in fasting serum glucose with changes in predicted fatty liver score

**DOI:** 10.1038/s41598-023-37194-x

**Published:** 2023-06-26

**Authors:** Saemi Han, Seogsong Jeong, Joseph C. Ahn, Yoosun Cho, Seulggie Choi, Sun Jae Park, Kyae Hyung Kim, Gyeongsil Lee, Joung Sik Son, Sang Min Park

**Affiliations:** 1 Department of Medicine, Seoul National University College of Medicine, Seoul, South Korea; 2 Department of Biomedical Sciences, Seoul National University Graduate School, Seoul, South Korea; 3 Department of Biomedical Informatics, CHA University School of Medicine, Seongnam, South Korea; 4 Division of Gastroenterology and Hepatology, Mayo Clinic, Rochester, MN USA; 5 Total Healthcare Center, Kangbuk Samsung Hospital, Sungkyunkwan University School of Medicine, Seoul, South Korea; 6 Department of Family Medicine, Seoul National University Hospital, Seoul, South Korea; 7 KS Health Link Inst. and Life Clinic, Seoul, South Korea; 8 Department of Internal Medicine, Hallym University Sacred Heart Hospital, Anyang, South Korea

**Keywords:** Non-alcoholic fatty liver disease, Lifestyle modification, Preventive medicine, Metabolic syndrome

## Abstract

Major post-cessation metabolic changes include weight gain and hyperglycemia. However, the association of post-cessation change in fasting serum glucose (FSG) with risk of fatty liver remains unclear. A total of 111,106 participants aged 40 and above who underwent health screening at least once in two examination periods were extracted from the Korean National Health Insurance Service-National Sample Cohort. Fatty liver status was evaluated using the Korean National Health and Nutrition Examination Survey nonalcoholic fatty liver disease (K-NAFLD) score. Linear and logistic regression were used to calculate the adjusted mean (aMean) and adjusted odds ratio (aOR) with 95% confidence intervals. Compared to stable (aMean 0.10; 95% CI 0.03–0.18) and decline (aMean − 0.60; 95% CI − 0.71 to 0.49) groups, FSG elevation (aMean 1.28; 95% CI 1.16–1.39) was associated with higher K-NAFLD score even within different body mass index change groups. Risk of fatty liver was significantly reduced among participants with stable (aOR 0.38; 95% CI 0.31–0.45) and declined (aOR 0.17; 95% CI 0.13–0.22) FSG levels after smoking cessation compared to FSG elevation group. This study suggests that quitters with elevated FSG are associated with higher NAFLD risk and may benefit from careful monitoring of FSG levels and management of other cardiovascular risk factors.

## Introduction

Cigarette smoking is associated with many adverse health outcomes such as various forms of cancer, cardiovascular disease, diabetes and more^1–3^. Therefore, smoking cessation comes highly recommended as a modifiable behavioral change that can improve various health outcomes. However, smoking cessation is also associated with a significant increase in weight and impaired fasting serum glucose (FSG) levels, which are risk factors for many metabolic-related diseases^4–6^. Many researchers then asked the question of whether post-cessation metabolic changes such as weight gain and hyperglycemia attenuate the benefits of quitting.

Hu et al.^7^ investigated post-cessation weight gain and type 2 diabetes risk in addition to cardiovascular and all-cause mortality and concluded that there was a short-term increase in type 2 diabetes risk which was proportional to the gain in weight while cardiovascular and all-cause mortality were decreased. Similarly, we previously found that regardless of the post-cessation change in FSG levels, the benefits of cardiovascular disease risk were not attenuated^8^.

Despite the abundance of research investigating the association between post-cessation metabolic changes and CVD incidence, CVD-related mortality, and all-cause mortality, there is a lack of research looking at fatty liver risk as an outcome. Fatty liver risk increases with BMI increase and impaired FSG levels, both of which are post-cessation changes commonly experienced by quitters^9,10^. In addition, cases of non-alcoholic fatty liver disease (NAFLD) are increasing, and the burden of the disease is predicted to be high with advanced NAFLD cases leading to steatohepatitis, fibrosis, and in extreme cases, liver failure and mortality^9,11^. Therefore, it has become clinically more important to understand risks factors associated with the disease.

There has been a study looking at NAFLD reoccurrence, where Nakanishi et al.^12^ found that smoking cessation increased the reoccurrence of NAFLD, likely due to the weight gain associated with quitting. The general logic seems to be that quitting leads to weight gain and then FSG increase which then results in increased risk of fatty liver. However, no direct studies have been carried out to show such flow of events. Our study attempted to investigate this association by calculating fatty liver risk in quitters, continual smokers, never-smokers, and ex-smokers and analyzed the difference based on the change in participant BMI and FSG levels. To measure fatty liver risk, we used the K-NAFLD scoring system, which was derived to predict fatty liver, metabolic dysfunction, and CVD risk^13,14^.

## Methods

### Study population

The Korean National Health Insurance Service (NHIS) provides obligatory medical insurance for Korean citizens, and biannual health screening is carried out in individuals aged 40 or more^15^. This study used the NHIS-National Sample Cohort, which is a random sampling of the NHIS database built to represent the entire population. This database includes sociodemographic data, medical history, hospitalization and outpatient department visit, serological characteristics, drug prescriptions, and lifestyle behaviors.

**Figure 1:**
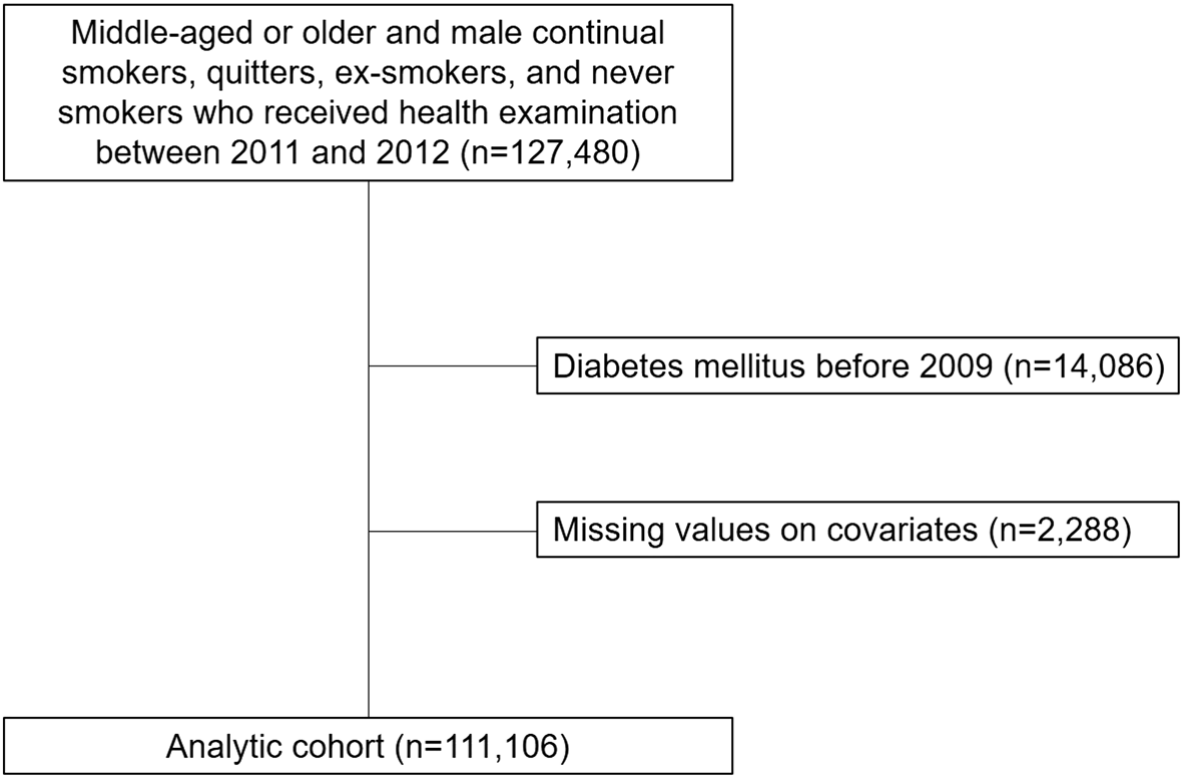
Flow diagram for the inclusion of study participants.

The study population is consisted of middle-aged or older men who are either continual smokers, quitters, ex-smokers, or never smokers. To define smoking status, participants who underwent health screening at least once in each two period (2009 to 2010 and 2011 to 2012) were extracted (n = 127,480; Fig. 1). Therefore, the cessation period for quitters in this study varies from one year (2010–2011) to three years (2009–2012). To limit hyperglycemia to non-diabetic participants, those with a history of diabetes mellitus before 2009 (n = 14,086) were excluded. In addition, participants with missing values on covariates (n = 2288) were excluded. Finally, 111,106 male continual smokers, quitters, ex-smokers, and never smokers aged at least 40 comprised the analytical cohort. This study adheres to the principles stated within the Declaration of Helsinki and the Institutional Review Board of Seoul National University Hospital approved this study (E-2108-136-1246). The requirement for informed consents was waived by the Seoul National University Hospital Institutional Review Board since the NHIS-National Sample Cohort was provided for research purposes in an anonymized form according to strict confidentiality guidelines.

### K-NAFLD score and NAFLD

The K-NAFLD score was calculated as follows: 0.913 $\times$ sex (1, if men; 2, if women) + 0.089 $\times$ waist circumference + 0.032 $\times$ (systolic blood pressure + FSG) + triglyceride $\times$ 0.007 + alanine aminotransferase $\times$ 0.105–20.929^13^. The score was calculated twice, first using the results of health screening that is carried out in 2009 to 2010 (first health screening) and second using the results from the second health screening carried out in 2011 to 2012. The change in K-NAFLD score was calculated by subtracting first K-NAFLD score from second K-NAFLD score. In addition, the presence of NAFLD was defined as the second K-NAFLD score > 0.884 in accordance with the suggested cut-off value^13^.

### Smoking groups

The NHIS-National Sample Cohort provides smoking status data in a ternary form (never smoker, past smoker, and current smoker). Continual smoker, quitter, ex-smoker, and never smoker were defined according to the following change in smoking status between two health screening periods: current smoker to current smoker, current smoker to past smoker, past smoker to past smoker, and never smoker to never smoker, respectively. To clarify further, those who were ex-smoker in both health screening periods were defined as ex-smokers, whereas those who were current smoker at the first health screening but ex-smoker at the second health screening were defined as quitters. Therefore, the term quitter refers to a recent quitter, whereas ex-smoker refers to quitters with a relatively longer period of smoking cessation.

### FSG groups

Change in FSG was classified as FSG elevation, FSG stable, and FSG decline according to 25th percentile and 75th percentile of change in FSG level. FSG increase of more than 12 mg/dL and FSG decrease of less than − 7 mg/dL were defined as FSG elevation and FSG decline, respectively. In addition, no FSG elevation or FSG decline was considered FSG stable.

### Key variables

The following variables were included for the adjusted analyses: age (continuous; years), household income (categorical; upper half and lower half), systolic blood pressure (continuous; mmHg), FSG (continuous; mg/dL), change in BMI (continuous; kg/m^2^), triglyceride (continuous; mg/dL), alcohol consumption (categorical; none, 1–4 times/week, and ≥ 5 times/week), moderate-to-vigorous physical activity (categorical; none, 1–4 times/week, and ≥ 5 times/week), and Charlson comorbidity index (categorical; 0, 1, and ≥ 2).

### Statistical analysis

The primary outcome was change in K-NAFLD score, and the secondary outcome was NAFLD defined using the K-NAFLD score in 2011–2012 period. Descriptive characteristics were presented as median (interquartile range; IQR). *P* values for the difference in median values was calculated using the Kruskal–Wallis H test. T tests were used in evaluation of statistical significance for difference in mean K-NAFLD scores. Linear regression was used in evaluating between-group difference of adjusted change in K-NAFLD score, more specifically, linear regression was used for two groups and the *P* value was calculated independently. The result was presented as adjusted mean (aMean) with 95% confidence interval (CI). The following adjustment models were used when calculating the aMean change in K-NAFLD score: age, household income, systolic blood pressure, change in BMI, triglyceride, and baseline K-NAFLD score (model A), and alcohol consumption, moderate-to-vigorous physical activity, Charlson comorbidity index, and variables included in model A (model B). To control the change in BMI when evaluating the association of FSG status with change in K-NAFLD score, participants were stratified into three groups: no BMI change (change in BMI ranging between − 1.0 kg/m^2^ and + 1.0 kg/m^2^, BMI gain (change in BMI greater than + 1.0 kg/m^2^), and BMI loss (BMI loss of more than 1.0 kg/m^2^). The risk of fatty liver was evaluated using the multivariable logistic regression after excluding participants with fatty liver during the 2009–2010 period (n = 94,903). All statistical analyses were carried out using SAS (v9.4; SAS Institute Inc.).

## Results

### Descriptive characteristics

Descriptive characteristics of the study population are shown in Table 1. There were 111,106 men with a median age of 54 (IQR 50–61), including 32,614 continual smokers (29.4%), 6765 quitters (6.1%), 34,241 ex-smokers (30.8%), and 37,486 never smokers (33.7%). A majority of the participants belonged to the upper-half group based on household income (n = 80,289; 72.3%). The median BMI and waist circumference were 24.0 kg/m^2^ (IQR 22.2–25.8 kg/m^2^) and 84 cm (IQR 80–89 cm). Additionally, 38,564 participants (34.7%) had no comorbidities.

**Table 1:** Baseline characteristics of the participants.

Characteristic	Participant (n = 111,106)
Age, years	54 (50–61)
Household income, upper half, n (%)	80,289 (72.3)
Body mass index, kg/m^2^	24.0 (22.2–25.8)
Waist circumference, cm	84 (80–89)
Systolic blood pressure, mmHg	125 (117–135)
Fasting serum glucose, mg/dL	96 (88–106)
Total cholesterol, mg/dL	195 (173–219)
Triglyceride, mg/dL	124 (87–181)
Aspartate aminotransferase, IU/L	23 (17–32)
Alanine aminotransferase, IU/L	25 (21–30)
**γ**-glutamyl transferase, IU/L	32 (22–53)
Smoking status, n (%)	
Continual smoker	32,614 (29.4)
Quitter	6765 (6.1)
Ex-smoker	34,241 (30.8)
Never smoker	37,486 (33.7)
Moderate-to-vigorous physical activity, n (%)	
None	42,531 (38.3)
1–4 time/week	30,667 (27.6)
≥ 5 time/week	37,908 (34.1)
Alcohol consumption, n (%)	
None	39,261 (35.3)
1–4 time/week	62,880 (56.6)
≥ 5 time/week	8965 (8.1)
Charlson comorbidity index, n (%)	
0	38,564 (34.7)
1	35,842 (32.3)
≥ 2	36,700 (33.0)

Data are median (interquartile range) unless indicated otherwise.

### Association of change in FSG and smoking status on change in K-NAFLD score

The median change in K-NAFLD scores were 0.83, 0, and − 0.82 in FSG elevation, FSG stable, and FSG decline groups, respectively (Supplementary Table 1). In the fully adjusted model, the aMean change in K-NAFLD scores were significantly higher for the FSG elevation group (aMean, 0.97; 95% CI 0.94–1.00) compared to the FSG stable (aMean, − 0.10; 95% CI − 0.12 to − 0.08) and the FSG decline group (aMean − 0.83; 95% CI − 0.85 to − 0.80). In addition, the aMean K-NAFLD score was lowest for never smokers (aMean, − 0.11; 95% CI − 0.13 to − 0.08), followed by continual smokers (aMean − 0.10; 95% CI − 0.13 to − 0.08), ex-smokers (aMean − 0.07; 95% CI − 0.09 to − 0.04), and quitters (aMean 0.20; 95% CI 0.14–0.25; Supplementary Table 2).

### Combined association of change in FSG and smoking status with change in K-NAFLD score

In a composite term of FSG and smoking, quitters in the FSG elevation (post-cessation hyperglycemia) group were set as the reference, and the aMean change in K-NAFLD scores was compared with other groups (Table 2). aMean change in K-NAFLD scores were increased in all FSG elevation groups and decreased in all FSG decline groups. The largest increase was found in quitters with FSG elevation (aMean 1.28; 95% CI 1.16–1.39), followed by continual smokers with FSG elevation (aMean, 1.02) and quitters with FSG stable (aMean, 0.10).

**Table 2:** Association of smoking status and change in FSG on change in K-NAFLD score among continual smokers or quitters.

	Participant (%)	Median (IQR)	Mean (SD)	*P* value	aMean (95% CI)	*P* value
Quitter						
FSG elevation	1578 (1.4)	1.23 (0.15–2.50)	1.45 (3.61)	Reference	1.28 (1.16–1.39)	Reference
FSG stable	3550 (3.2)	0.29 (− 0.77 to 1.34)	0.30 (2.54)	< 0.001	0.10 (0.03–0.18)	< 0.001
FSG decline	1637 (1.5)	− 0.51 (− 1.74 to 0.57)	− 0.61 (3.15)	< 0.001	− 0.60 (− 0.71 to 0.49)	< 0.001
Continual smoker						
FSG elevation	6845 (6.2)	0.87 (− 0.25 to − 2.04)	0.97 (3.27)	< 0.001	1.02 (0.97–1.07)	0.003
FSG stable	17,170 (15.5)	− 0.01 (− 0.99 to 0.95)	− 0.02 (2.56)	< 0.001	− 0.14 (− 0.18 to 0.11)	< 0.001
FSG decline	8599 (7.7)	− 0.87 (− 2.08 to 0.19)	− 1.04 (3.08)	< 0.001	− 0.92 (− 0.97 to 0.87)	< 0.001

*P* values for means calculated using the Kruskal–Wallis H test.

aMeans and *P* values calculated using the linear regression after adjustments for age, household income, baseline fasting serum glucose, change in body mass index, triglycerides, alcohol consumption, moderate-to-vigorous physical activity, Charlson comorbidity index, and baseline K-NAFLD score.

*K-NAFLD* Korean nutritional health and nutrition examination survey nonalcoholic fatty liver disease, *IQR *interquartile range, *SD* standard deviation, *aMean* adjusted mean.

### Combined association of change in FSG and smoking status with change in K-NAFLD score according to the change in BMI

**Figure 2:**
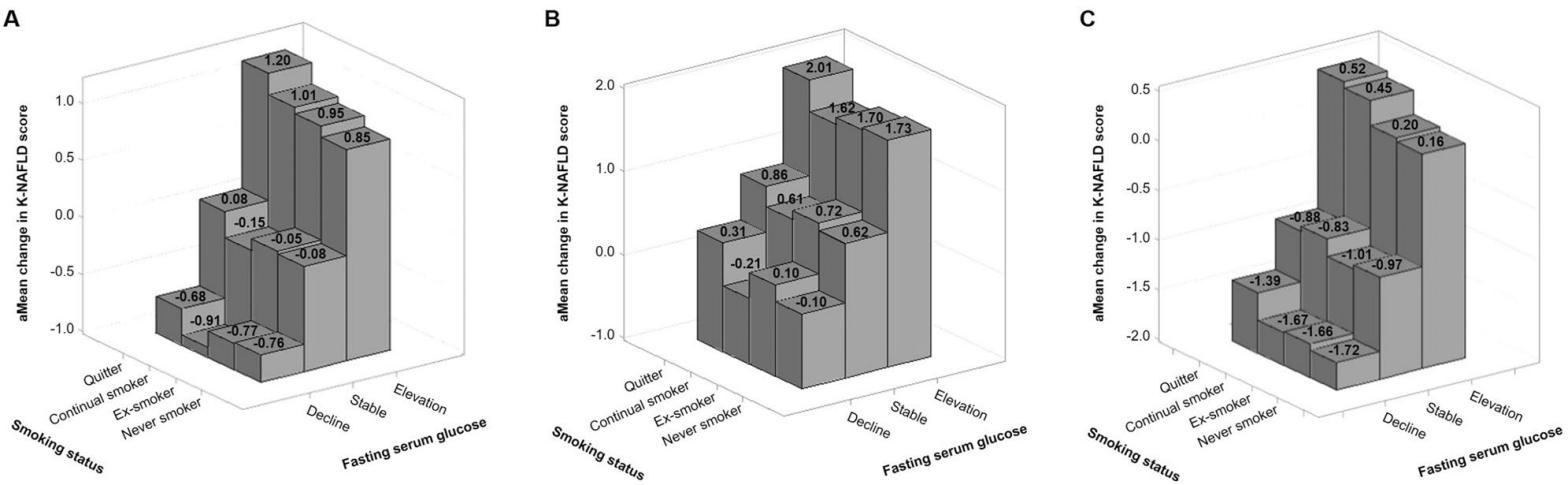
Association of smoking status and change in fasting serum glucose with change in K-NAFLD score. Adjusted mean change in K-NAFLD score calculated using linear regression after adjustments for age, household income, systolic blood pressure, triglycerides, alcohol consumption, moderate-to-vigorous physical activity, and Charlson comorbidity index. (**A**) Participants with no BMI change. (**B**) Participants with BMI gain. (**C**) Participants with BMI loss.

Since the K-NAFLD score, FSG level, and fatty liver are significantly affected by change in BMI, we assessed the association of change in FSG and smoking status with change in K-NAFLD score in participants with no BMI change, BMI gain, and BMI loss, respectively. Among participants with no BMI change, the difference in aMean change in K-NAFLD score between quitters with FSG elevation and FSG decline was 1.88 (Fig. 2A). As for participants with BMI gain, the difference in an aMean change in K-NAFLD score was 1.70 between quitters with FSG elevation and FSG decline (Fig. 2B). In addition, the difference between aMean change in K-NAFLD scores was 1.91 for participants with BMI loss (Fig. 2C).

### Change in components of the K-NAFLD score according to change in smoking status and fasting serum glucose level

We then compared change in the components of K-NAFLD score, including alanine aminotransferase, waist circumference, systolic blood pressure, and triglyceride, to confirm which component is more sensitive to change in the FSG and smoking status groups (Supplementary Fig. 1). There was no significant difference when looking at change in alanine aminotransferase level among quitters with FSG elevation compared to quitters with a stable FSG and FSG decline. As for waist circumference and systolic blood pressure, participants with FSG decline showed a significant decrease compared to quitters with FSG elevation. In addition, triglyceride significantly increased in quitters with FSG elevation compared to quitters with a stable FSG and FSG decline.

### Stratified analysis on comparison of change in aMean K-NAFLD score according to change in FSG

In stratified analyses, all subgroups showed significantly increased aMean K-NAFLD score in the FSG elevation group, whereas the score slightly decreased in the FSG stable group while significant decreases were found for the FSG decline group (Table 3). There was no subgroup with significant differences with the primary findings found in the overall study population.

**Table 3:** Stratified analysis on comparison of adjusted mean K-NAFLD according to FSG elevation or without FSG elevation.

	FSG elevation	FSG stable	FSG decline	*P* value^a^	*P* value^b^	*P* value^c^
Age						
≥ 60 years	0.88 (0.83–0.94)	− 0.16 (− 0.20–0.13)	− 0.91 (− 0.96–0.86)	< 0.001	< 0.001	< 0.001
< 60 years	1.00 (0.96–1.04)	− 0.08 (− 0.10–0.05)	− 0.80 (− 0.83–0.76)	< 0.001	< 0.001	< 0.001
Household income						
Upper half	0.95 (0.92–0.99)	− 0.10 (− 0.12–0.08)	− 0.81 (− 0.84–0.78)	< 0.001	< 0.001	< 0.001
Lower half	1.00 (0.94–1.06)	− 0.12 (− 0.16–0.09)	− 0.88 (− 0.94–0.83)	< 0.001	< 0.001	< 0.001
Obesity						
BMI ≥ 25 kg/m^2^	0.92 (0.87–0.97)	− 0.25 (− 0.29–0.22)	− 1.00 (− 1.05–0.95)	< 0.001	< 0.001	< 0.001
BMI < 25 kg/m^2^	0.98 (0.94–1.02)	− 0.01 (− 0.04–0.01)	− 0.70 (− 0.73–0.66)	< 0.001	< 0.001	< 0.001
MVPA						
None	0.96 (0.91–1.01)	− 0.13 (− 0.16–0.10)	− 0.91 (− 0.96–0.87)	< 0.001	< 0.001	< 0.001
1–4 time/week	0.88 (0.82–0.94)	− 0.12 (− 0.16–0.09)	− 0.81 (− 0.86–0.75)	< 0.001	< 0.001	< 0.001
≥ 5 time/week	1.06 (1.00–1.11)	− 0.04 (− 0.08–0.01)	− 0.74 (− 0.79–0.69)	< 0.001	< 0.001	< 0.001
Alcohol consumption						
None	0.95 (0.91–1.02)	− 0.13 (− 0.17–0.10)	− 0.80 (− 0.85–0.75)	< 0.001	< 0.001	< 0.001
1–4 time/week	0.96 (0.92–1.00)	− 0.08 (− 0.10–0.05)	− 0.81 (− 0.85–0.77)	< 0.001	< 0.001	< 0.001
≥ 5 time/week	1.06 (0.95–1.18)	− 0.15 (− 0.22–0.07)	− 1.04 (− 1.15–0.94)	< 0.001	< 0.001	< 0.001
Charlson comorbidity index						
0	0.99 (0.94–1.04)	− 0.07 (− 0.10–0.03)	− 0.82 (− 0.86–0.77)	< 0.001	< 0.001	< 0.001
1	0.95 (0.90–1.01)	− 0.11 (− 0.15–0.08)	− 0.82 (− 0.87–0.77)	< 0.001	< 0.001	< 0.001
≥ 2	0.96 (0.90–1.02)	− 0.13 (− 0.16–0.09)	− 0.85 (− 0.90–0.79)	< 0.001	< 0.001	< 0.001

Data are adjusted median (95% confidence interval) calculated using the linear regression after adjustments for age, household income, systolic blood pressure, change in body mass index, triglyceride, baseline Korean Nutritional Health and Nutrition Examination Survey nonalcoholic fatty liver disease score, alcohol consumption, moderate-to-vigorous physical activity, and Charlson comorbidity index.

^a^*P* values between FSG elevation and FSG stable groups.

^b^*P* values between FSG elevation and FSG decline groups.

^c^*P* values between FSG stable and FSG decline groups.

*FSG* fasting serum glucose, *BMI* body mass index, *MVPA* moderate-to-vigorous physical activity.

### Association of change in FSG and smoking status with risk of NAFLD among participants without NAFLD at baseline

To evaluate the risk of fatty liver according to change in FSG, participants with NAFLD at baseline (2009–2010) were excluded. The proportion of fatty liver at the second health screening period (2011–2012) was 15.9% (n = 2873), 6.5% (n = 54,055), and 5.2% (n = 22,757) for FSG elevation, stable, and decline, respectively (Supplementary Table 3). In addition, the FSG elevation group (adjusted odds ratio [aOR] 3.01; 95% CI 2.84–3.18) and the FSG decline group (aOR 0.51; 95% CI 0.47–0.55) showed significantly higher and lower risks of fatty liver in the fully adjusted model compared to the FSG stable group.

After stratification according to change in FSG and smoking status, all subgroups revealed lower risk of fatty liver compared to quitters with FSG elevation (Table 4). The aOR was lowest in never smokers with FSG decline, followed by continual smokers with FSG decline, ex-smokers with FSG decline, quitters with FSG decline, never smokers with stable FSG, continual smoker with stable FSG, ex-smokers with stable FSG, quitters with stable FSG, never smokers with FSG elevation, continual smokers with FSG elevation, ex-smokers with FSG elevation, and quitters with FSG elevation.

**Table 4:** Association of change in smoking status and FSG with risk of fatty liver.

	Participant (%)	Event (%)	Univariable analysis		Multivariable analysis	
			OR (95% CI)	*P* value	aOR (95% CI)	*P* value
FSG elevation						
Continual smoker	5630 (5.9)	956 (17.0)	0.77 (0.66–0.89)	< 0.001	0.83 (0.71–0.97)	0.019
Quitter	1361 (1.4)	286 (21.0)	1.00 (Reference)		1.00 (Reference)	
Ex-smoker	5140 (5.4)	821 (16.0)	0.71 (0.62–0.83)	< 0.001	0.84 (0.72–0.99)	0.037
Never smoker	5960 (6.3)	810 (13.6)	0.59 (0.51–0.69)	< 0.001	0.81 (0.69–0.95)	0.008
FSG stable						
Continual smoker	15,000 (15.8)	1041 (6.9)	0.28 (0.24–0.32)	< 0.001	0.27 (0.23–0.32)	< 0.001
Quitter	3096 (3.3)	312 (10.1)	0.42 (0.35–0.50)	< 0.001	0.38 (0.31–0.45)	< 0.001
Ex-smoker	16,963 (17.9)	1097 (6.5)	0.26 (0.23–0.30)	< 0.001	0.28 (0.24–0.32)	< 0.001
Never smoker	18,996 (20.0)	1046 (5.5)	0.22 (0.19–0.25)	< 0.001	0.27 (0.23–0.31)	< 0.001
FSG decline						
Continual smoker	6691 (7.1)	384 (5.7)	0.23 (0.19–0.27)	< 0.001	0.14 (0.12–0.17)	< 0.001
Quitter	1289 (1.4)	97 (7.5)	0.31 (0.24–0.39)	< 0.001	0.17 (0.13–0.22)	< 0.001
Ex-smoker	6959 (7.3)	369 (5.3)	0.21 (0.18–0.25)	< 0.001	0.15 (0.12–0.18)	< 0.001
Never smoker	7818 (8.2)	329 (4.2)	0.17 (0.14–0.20)	< 0.001	0.13 (0.11–0.16)	< 0.001

OR calculated using logistic regression. aOR calculated after adjustments for age, household income, systolic blood pressure, baseline fasting serum glucose, change in body mass index, triglycerides, alcohol consumption, moderate-to-vigorous physical activity, and Charlson comorbidity index.

*FSG* fasting serum glucose, *OR* odds ratio, *CI* confidence interval, *aOR* adjusted odds ratio.

## Discussion

Using data from the Korean NHIS, we extracted 111,106 male subjects aged 40 and over, FSG elevation was associated with increased K-NAFLD score even after adjustments for key variables and stratification according to change in smoking and BMI statuses. Close monitoring of FSG seems necessary for quitters to reduce the K-NAFLD score, thus implying that management of FSG through medication or lifestyle modifications may be promising in reducing fatty liver incidence.

Takenaka et al.^16^, investigated the association between smoking cessation and NAFLD risk, showing that current smoking is associated with NAFLD incidence and higher pack-years were associated with more severe disease. Another more recent paper by Jang et al.^17^, divided individuals who have quit smoking based on years since cessation in multiples of 10 years and showed an association between smoking cessation and NAFLD incidence with current smokers having a greater risk of NAFLD than non-smokers. While NAFLD may perhaps decrease in the long run after smoking cessation, we divided ex-smokers with quitters to distinguish the risk between those who have recently quit and have quit for a longer period of time. We also wanted to highlight that not all quitters are the same—the same way some quitters gain weight after cessation and others don’t. It is well accepted that weight gain and risk of metabolic disease increases after smoking cessation. The value in this study is that it attempts to divide quitters, a group already known to have metabolic changes, into groups that allows us to better find those at risk of NAFLD after cessation. In this study, FSG elevation showed a strong association with NAFLD risk regardless of weight gain status, perhaps indicating that changes in FSG levels can be a method of determining those with higher risk of NAFLD after smoking cessation.

There are many studies that have investigated the impact of smoking on insulin resistance and glucose metabolism, showing that chronic smoking increases insulin resistance and studies that have looked at the mechanism behind such insulin resistance such as cortisol release and pancreatic β-cell damage^18–30^. Though there is literature showing that an increase in fasting glucose levels follows smoking cessation, the mechanism in which post-cessation hyperglycemia occurs is unknown. Though Oba et al.^21^ broadly suggest that smoking cessation exposes the pancreatic damage occurred by nicotine while smoking, the exact mechanism in which increased insulin resistance and impaired fasting glucose occurs after cessation is unclear.

There are multiple mechanisms that can explain the increased risk of fatty liver due to an increase in fasting serum glucose levels. An excess of blood sugars, also known as glucotoxicity, is known to cause damage and inflammation and the consequences on the liver can be great. First, long-term hyperglycemia leads to oxidative stress and inflammation, which can lead to increased liver disease and the apoptosis of hepatocytes^22–24^. Glucotoxicity can directly cause inflammation by causing increased TNFα transcription and the upregulation of NFκβ, a pro-inflammatory pathway^25^. Hyperglycemia causes oxidative stress by activating the production of reactive oxygen species (ROS) and toll-like receptor (TLR) signaling, which then promote an inflammatory response^26^. Second, such oxidative stress and inflammation can cause beta cell damage and insulin resistance, leading to the accumulation of fat in the liver, known as steatosis, which has been shown to cause damage in the mitochondria and endoplasmic reticulum through pathways involving JNK activation which then upregulate genes that are pro-apoptotic like PUMA^24,27–29^. The glucotoxicity and lipotoxicity combined and the mechanisms that follow contribute to steatosis and inflammation, which are hallmarks of fatty liver^30^.

In this study, we subdivided participants based on change in BMI and smoking status, yet the data exhibited a significant change in K-NAFLD score according to FSG elevation, stable, or decline status. The K-NAFLD score increase reflects not only an increased risk of fatty liver but also metabolic risk factors and insulin resistance^13^. Especially with an increase in insulin resistance, the effects of hyperglycemia become amplified, and a vicious cycle can occur where fatty liver risk increases^31^. Since the K-NAFLD score reflects not just fatty liver disease risk but other metabolic dysfunctions and risk factors, we previously found the score to be directly proportional to the risk of CVD^14^. Even in patients with no metabolic dysfunctions, a lower K-NAFLD score was associated with lowered CVD risk. Based on the results from this study showing that quitters, especially those with elevated FSG, have the highest increase in K-NAFLD score, there may be a benefit in more carefully monitoring fatty liver incidence and management of other cardiovascular risk factors in these individuals during health check-ups.

This study was able to include over 100,000 male participants who were continual smokers, quitters, ex-smokers, and never-smokers. The participants only included non-diabetic individuals to be able to accurately assess the impact of hyperglycemia on K-NAFLD risk. Also, since one of the most important changes that occur after smoking cessation is weight gain, which could confound the results, we included an analysis where participants were divided into groups based on BMI weight change and looked at FSG change in each group.

However, this study also had limitations. Information included within the database was all we had access to and therefore, we were not able to look at HbA1c or oral glucose tolerance test (OGTT) measurements, which are reflections of longer-term blood glucose levels and insulin resistance status, respectively. Though we were able to address this limitation by excluding those with a history of diabetes, there were also other lifestyle changes such as eating habits that could impact fatty liver risk, which we were unable to reflect. This inability to reflect lifestyle changes may explain the result where quitters sometimes show a higher risk of fatty liver compared to continual smokers, ex-smokers, and never-smokers even when BMI and FSG levels were stable or decreased. The smoker status of participants was not biochemically validated, but rather self-reported during their health examination. Therefore, there is a risk that smoker status of participants does not correctly reflect the actual smoking status—a limitation associated with health examination questionnaires. We also were unable to reflect intensity of smoking in this study; such data would have allowed us to further categorize ex-smokers and can be considered in a follow-up study. Lastly, the onset of fatty liver is a chronic process, but since the study only looked at changes occurred in two years, we were unable to investigate the long-term effect of changes in fasting serum glucose and smoking status. However, since we looked at the change in K-NAFLD score as a continuous variable, we could predict long-term trends based on our results.

An increase in fasting serum glucose levels is generally followed by cigarette cessation. However, it was unclear whether such changes in fasting serum glucose levels based on smoking status impact risk of fatty liver disease. Along the lines of previous studies, the quitter group showed the highest increase in FSG levels. The increase in FSG levels were associated with a higher risk in fatty liver. The results were consistent even in different groups stratified according to change in BMI. The general understanding is that weight gain following smoking cessation causes hyperglycemia, insulin resistance and other metabolic risk factors that increases diabetes risk. However, this study suggests regardless of weight gain status, hyperglycemia has a direct association with fatty liver risk. Therefore, not only should weight gain be monitored and controlled, but surveillance of FSG also shows potential in reducing the societal disease burden of fatty liver.

## Supplementary Information

Supplementary Information.

## Data Availability

The dataset utilized and analyzed in this dataset was accessed from the National Health Insurance Service. The authors cannot publically share the database utilized due to reasons concerning data protection and security. However, any interested researchers are able to access the same anonymized database by following access procedures and guidelines put forth by the Korean National Health Insurance Service. Please refer to the following website (http://nhiss.nhis.or.kr) for more information.

## References

[1] Saha SP, Bhalla DK, Whayne TF, Gairola C (2007). Cigarette smoke and adverse health effects: An overview of research trends and future needs. Int. J. Angiol..

[2] Ambrose JA, Barua RS (2004). The pathophysiology of cigarette smoking and cardiovascular disease: An update. J. Am. Coll. Cardiol..

[3] Willi C, Bodenmann P, Ghali WA, Faris PD, Cornuz J (2007). Active smoking and the risk of type 2 diabetes: A systematic review and meta-analysis. JAMA.

[4] Harris KK, Zopey M, Friedman TC (2016). Metabolic effects of smoking cessation. Nat. Rev. Endocrinol..

[5] Stein JH, Asthana A, Smith SS (2014). Smoking cessation and the risk of diabetes mellitus and impaired fasting glucose: Three-year outcomes after a quit attempt. PLoS ONE.

[6] Lee SS, Seo JS, Kim SR (2011). The changes of blood glucose control and lipid profiles after short-term smoking cessation in healthy males. Psychiatry Investig..

[7] Hu Y, Zong G, Liu G (2018). Smoking cessation, weight change, type 2 diabetes, and mortality. N. Engl. J. Med..

[8] Choi S, Kim K, Chang J (2017). Effect of post-cessation hyperglycemia on cardiovascular disease and mortality among middle-aged men: An eight-year longitudinal study. Sci. Rep..

[9] Cotter TG, Rinella M (2020). Nonalcoholic fatty liver disease 2020: The state of the disease. Gastroenterology.

[10] Zou Y, Yu M, Sheng G (2020). Association between fasting plasma glucose and nonalcoholic fatty liver disease in a nonobese Chinese population with normal blood lipid levels: A prospective cohort study. Lipids Health Dis..

[11] Mundi MS, Velapati S, Patel J, Kellogg TA, Abu Dayyeh BK, Hurt RT (2020). Evolution of NAFLD and its management. Nutr. Clin. Pract..

[12] Nakanishi N, Hashimoto Y, Okamura T (2021). A weight regain of 1.5 kg or more and lack of exercise are associated with nonalcoholic fatty liver disease recurrence in men. Sci. Rep..

[13] Jeong S, Kim K, Chang J (2020). Development of a simple nonalcoholic fatty liver disease scoring .system indicative of metabolic risks and insulin resistance. Ann. Transl Med..

[14] Jeong S, Oh YH, Choi S (2022). Metabolic dysfunction-associated fatty liver disease better predicts incident cardiovascular disease. Gut Liver.

[15] Cheol Seong S, Kim YY, Khang YH (2017). Data resource profile: The national health information database of the national health insurance service in South Korea. Int. J. Epidemiol..

[16] Takenaka H, Fujita T, Masuda A, Yano Y, Watanabe A, Kodama Y (2020). Non-alcoholic fatty liver disease is strongly associated with smoking status and is improved by smoking cessation in Japanese males: A retrospective study. Kobe J. Med. Sci..

[17] Jang YS, Joo HJ, Park YS, Park EC, Jang SI (2023). Association between smoking cessation and non-alcoholic fatty liver disease using NAFLD liver fat score. Front Public Health.

[18] Kirschbaum C, Wüst S, Strasburger CJ (1992). ‘Normal’ cigarette smoking increases free cortisol in habitual smokers. Life Sci..

[19] Artese A, Stamford BA, Moffatt RJ (2017). Cigarette smoking: An accessory to the development of insulin resistance. Am. J. Lifestyle Med..

[20] Tong X, Chaudhry Z, Lee CC (2020). Cigarette smoke exposure impairs β-cell function through activation of oxidative stress and ceramide accumulation. Mol Metab..

[21] Oba S, Noda M, Waki K (2012). Smoking cessation increases short-term risk of type 2 diabetes irrespective of weight gain: The Japan public health center-based prospective study. PLoS ONE.

[22] Gilbert ER, Liu D (2012). Epigenetics: The missing link to understanding β-cell dysfunction in the pathogenesis of type 2 diabetes. Epigenetics.

[23] Harada S, Miyagi K, Obata T (2017). Influence of hyperglycemia on liver inflammatory conditions in the early phase of non-alcoholic fatty liver disease in mice. J. Pharm. Pharmacol..

[24] Mota M, Banini BA, Cazanave SC, Sanyal AJ (2016). Molecular mechanisms of lipotoxicity and glucotoxicity in nonalcoholic fatty liver disease. Metabolism.

[25] Vasiljević A, Bursać B, Djordjevic A (2014). Hepatic inflammation induced by high-fructose diet is associated with altered 11βHSD1 expression in the liver of Wistar rats. Eur. J. Nutr..

[26] Pahwa R, Jialal I (2016). Hyperglycemia induces toll-like receptor activity through increased oxidative stress. Metab. Syndr. Relat. Disord..

[27] Cerf ME (2013). Beta cell dysfunction and insulin resistance. Front. Endocrinol. (Lausanne).

[28] Malhi H, Bronk SF, Werneburg NW, Gores GJ (2006). Free fatty acids induce JNK-dependent hepatocyte lipoapoptosis. J. Biol. Chem..

[29] Cazanave SC, Elmi NA, Akazawa Y, Bronk SF, Mott JL, Gores GJ (2010). CHOP and AP-1 cooperatively mediate PUMA expression during lipoapoptosis. Am. J. Physiol. Gastrointest. Liver Physiol..

[30] Tilg H, Moschen AR (2010). Evolution of inflammation in nonalcoholic fatty liver disease: The multiple parallel hits hypothesis. Hepatology.

[31] Leclercq IA, Da Silva MA, Schroyen B, Van Hul N, Geerts A (2007). Insulin resistance in hepatocytes and sinusoidal liver cells: Mechanisms and consequences. J. Hepatol..

