# Supplementary material for: Association of post-smoking cessation changes in fasting serum glucose with changes in predicted fatty liver score
Source: Sci Rep. 2023 Jun 26;13:10300. doi: 10.1038/s41598-023-37194-x (PMC10293240; doi:10.1038/s41598-023-37194-x)
Supplement: Supplementary file 1 — Supplementary Information. [file 41598_2023_37194_MOESM1_ESM.docx]

**Supplementary Table 1. Association of FSG elevation on change in K-NAFLD score**

|  | FSG elevation  (n=21,424) | FSG stable  (n=61,214) | FSG decline  (n=28,468) | P_a-b_ | P_a-c_ | P_b-c_ |
| --- | --- | --- | --- | --- | --- | --- |
| Median (IQR) | 0.83 (-0.25-1.95) | 0.00 (-0.95-0.93) | -0.82 (-1.94-0.18) | <0.001 | <0.001 | <0.001 |
| Mean (SD) | 0.92 (3.01) | -0.02 (2.46) | -0.96 (2.78) | <0.001 | <0.001 | <0.001 |
| aMean (95% CI)^a^ | 0.98 (0.95-1.01) | -0.10 (-0.12--0.08) | -0.83 (-0.86--0.80) | <0.001 | <0.001 | <0.001 |
| aMean (95% CI)^b^ | 0.97 (0.94-1.00) | -0.10 (-0.12--0.08) | -0.83 (-0.85--0.80) | <0.001 | <0.001 | <0.001 |

P values for medians were calculated using the Kruskal-Wallis H test.

P values for means were calculated using the t test.

P values for adjusted means were calculated using the linear regression.

FSG elevation defined as change in FSG greater than +12 mg/dL in the second health screening (2011-2012) compared to the first health screening (2009-2010).

FSG stable defined as change in FSG ranging between -1.0 kg/m^2^ and +1.0 kg/m^2^ in the second health screening (2011-2012) compared to the first health screening (2009-2010).

FSG decline defined as FSG loss of more than 7 mg/dL in the second health screening (2011-2012) compared to the first health screening (2009-2010).

^a^Adjusted for age, household income, systolic blood pressure, change in body mass index, triglyceride, and baseline K-NAFLD score.

^b^Further adjusted for alcohol consumption, moderate-to-vigorous physical activity, and Charlson comorbidity index on the basis of model A.

Acronyms: K-NAFLD, Korean Nutritional Health and Nutrition Examination Survey nonalcoholic fatty liver disease score; FSG, fasting serum glucose; IQR, interquartile range; SD, standard deviation; aMean, adjusted mean; CI, confidence interval.

**Supplementary Table 2. Association of smoking status on change in K-NAFLD score**

|  | Continual smoker | Quitter | Ex-smoker | Never smoker | P_a-b_ | P_b-c_ | P_b-d_ |
| --- | --- | --- | --- | --- | --- | --- | --- |
| Median (IQR) | -0.06 (-1.18-1.05) | 0.32 (-0.85-1.48) | -0.09 (-1.13-0.93) | -0.10 (-1.11-0.93) | <0.001 | <0.001 | <0.001 |
| Mean (SD) | -0.08 (2.94) | 0.35 (3.05) | -0.14 (2.78) | -0.11 (2.46) | <0.001 | <0.001 | <0.001 |
| aMean (95% CI)^a^ | -0.09 (-0.12--0.07) | 0.20 (0.15-0.26) | -0.06 (-0.09--0.03) | -0.12 (-0.15--0.10) | <0.001 | <0.001 | <0.001 |
| aMean (95% CI)^b^ | -0.10 (-0.13--0.08) | 0.20 (0.14-0.25) | -0.07 (-0.09--0.04) | -0.11 (-0.13--0.08) | <0.001 | <0.001 | <0.001 |

P values for medians were calculated using the Kruskal-Wallis H test.

P values for means were calculated using the t test.

P values for adjusted means were calculated using the linear regression.

^a^Adjusted for age, household income, systolic blood pressure, change in body mass index, triglyceride, and baseline K-NAFLD.

^b^Further adjusted for alcohol consumption, moderate-to-vigorous physical activity, and Charlson comorbidity index on the basis of model A.

Acronyms: K-NAFLD, Korean Nutritional Health and Nutrition Examination Survey nonalcoholic fatty liver disease score; IQR, interquartile range; SD, standard deviation; aMean, adjusted mean; CI, confidence interval.

**
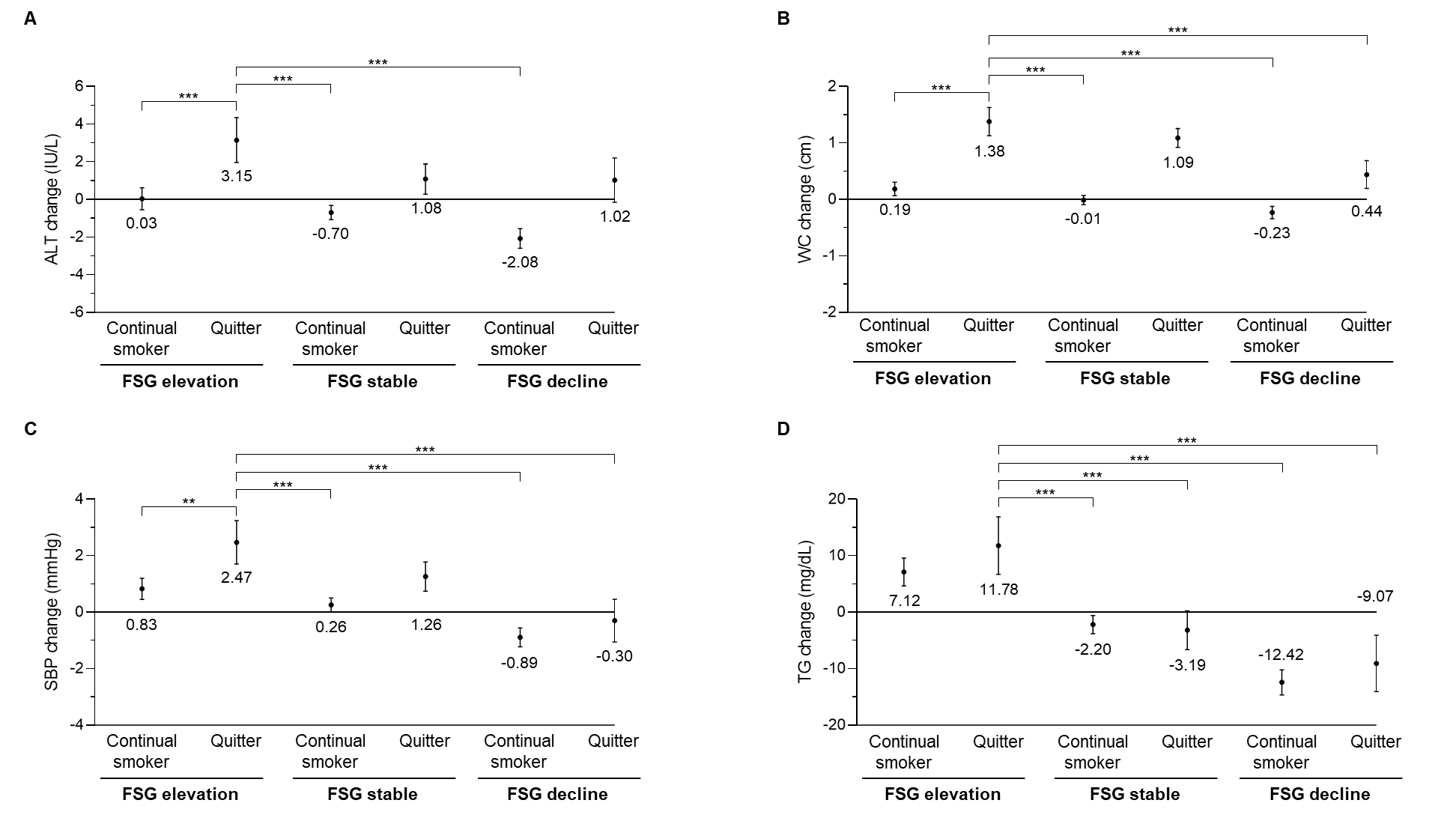
**

**Supplementary Figure 1. Change in components of the K-NAFLD score according to change in smoking status and fasting serum glucose level.** Adjusted means calculated using linear regression after adjustments for age, household income, alcohol consumption, moderate-to-vigorous physical activity, and Charlson comorbidity index. (A) Change in alanine aminotransferase. (B) change in waist circumference. (C) Change in systolic blood pressure. (D) Change in triglyceride.

**Supplementary Table 3.** **Association of post-cessation hyperglycemia with risk of fatty liver among participants without fatty liver at baseline**

|  | FSG elevation  (n=18,091) | FSG stable  (n=54,055) | FSG decline  (n=22,757) | P for trend |
| --- | --- | --- | --- | --- |
| Event (%) | 2,873 (15.9) | 3,496 (6.5) | 1,179 (5.2) |  |
| OR (95% CI) | 2.73 (2.59-2.88) | 1.00 (reference) | 0.79 (0.74-0.85) | <0.001 |
| aOR (95% CI)^a^ | 2.75 (2.61-2.90) | 1.00 (reference) | 0.80 (0.75-0.85) | <0.001 |
| aOR (95% CI)^b^ | 3.03 (2.87-3.20) | 1.00 (reference) | 0.51 (0.47-0.55) | <0.001 |
| aOR (95% CI)^c^ | 3.01 (2.84-3.18) | 1.00 (reference) | 0.51 (0.47-0.55) | <0.001 |

OR calculated using logistic regression.

^a^Adjusted for age.

^b^Further adjusted for household income, baseline fasting serum glucose, systolic blood pressure, change in body mass index, and triglycerides on the basis of model A.

^c^Further adjusted for alcohol consumption, moderate-to-vigorous physical activity, smoking status, and Charlson comorbidity index on the basis of model B.
